# A novel approach for US-guided central venous cannulation in phantom models: the oblique technique

**DOI:** 10.1186/2036-7902-6-S1-A22

**Published:** 2014-01-31

**Authors:** DE Morato, S Terp, M Chilstrom, CN Lam, M Menchine, T Mailhot

**Affiliations:** 1 Department of Emergency Medicine, LAC+USC Medical Center, Los Angeles, CA, USA; 2 Department of Emergency Medicine, University of Pittsburgh Medical Center, Pittsburgh, PA, USA

## Objective

Ultrasound (US) guided central vascular access is an indispensable skill for physicians. Recently, a novel approach to vascular cannulation, the oblique technique, has been described. This technique combines the advantages of the traditional (short axis, SA, and long axis, LA) approaches, while purportedly avoiding their pitfalls. This claim has not been well studied. We aimed to compare the three techniques to determine which is associated with the highest rate and fastest time to cannulation, and lowest rate of posterior vessel wall puncture (PVWP), a proxy for complications. We hypothesized that the oblique technique would be superior in all respects.

## Patients and methods

Sixty subjects including 35 Emergency Medicine residents and 25 senior medical students volunteered to participate. All subjects received standardized instruction on how to perform the three techniques prior to the study. The order of approach was randomized, and subjects were timed as they attempted to cannulate disposable vascular phantoms. Following the attempts, phantoms were deconstructed to assess outcomes.

## Results

Mean time to cannulation (in seconds) was not significantly different between the SA (38, 95% CI 30-46), LA (39, 95% CI 31-47), and oblique (31, 95% CI 28-35) techniques. There were no cannulation failures with the oblique technique (0%), compared to the SA (5%, 95% CI -1-10%) and LA (2%, 95% CI -2-5%) techniques. This difference did not reach significance. There was no significant reduction in the rate of PVWP with the oblique technique. We detected a higher likelihood of PVWP with SA (22%, 95% CI 11-32%) versus LA (5%, 95% CI -1-11%), or oblique (10%, 95% CI 2-18%) (Pearson chi2 8.2; df=2; p=0.02).

## Conclusion

The three techniques for US-guided central vessel cannulation are equivalent with respect to time to cannulation and rates of successful cannulation. The SA technique for US-guided central access is associated with a significant increase in PVWP when compared with LA or oblique approaches. This deserves further investigation, as it is the most frequently employed technique.

